# Transcriptional and post-transcriptional regulation of checkpoint genes on the tumour side of the immunological synapse

**DOI:** 10.1038/s41437-022-00533-1

**Published:** 2022-04-22

**Authors:** Paula Dobosz, Przemysław A. Stempor, Miguel Ramírez Moreno, Natalia A. Bulgakova

**Affiliations:** 1 Central Clinical Hospital of the Ministry of Interior Affairs and Administration in Warsaw, Warsaw, Poland; 2 SmartImmune Ltd, Cambridge, UK; 3 School of Biosciences and Bateson Centre, The University of Sheffield, Sheffield, UK

**Keywords:** Epigenetics, Gene regulation

## Abstract

Cancer is a disease of the genome, therefore, its development has a clear Mendelian component, demonstrated by well-studied genes such as *BRCA1* and *BRCA2* in breast cancer risk. However, it is known that a single genetic variant is not enough for cancer to develop leading to the theory of multistage carcinogenesis. In many cases, it is a sequence of events, acquired somatic mutations, or simply polygenic components with strong epigenetic effects, such as in the case of brain tumours. The expression of many genes is the product of the complex interplay between several factors, including the organism’s genotype (in most cases Mendelian-inherited), genetic instability, epigenetic factors (non-Mendelian-inherited) as well as the immune response of the host, to name just a few. In recent years the importance of the immune system has been elevated, especially in the light of the immune checkpoint genes discovery and the subsequent development of their inhibitors. As the expression of these genes normally suppresses self-immunoreactivity, their expression by tumour cells prevents the elimination of the tumour by the immune system. These discoveries led to the rapid growth of the field of immuno-oncology that offers new possibilities of long-lasting and effective treatment options. Here we discuss the recent advances in the understanding of the key mechanisms controlling the expression of immune checkpoint genes in tumour cells.

## Introduction

Over 19.3 million new cancer cases and almost 10 million cancer deaths occurred in 2020 worldwide (Sung et al. 2021). Thus, cancer remains a burning problem, especially in those countries, where society ageing is noticeable. As a disease of the genome, cancer development has a clear Mendelian component, demonstrated by several famous genes such as *BRCA1* and *BRCA2* in breast cancer risk (Smithers 1948; Murthy and Muggia 2019). However, it is also long appreciated that a single genetic variant might not be enough for cancer to develop, even if the risk of having cancer is significantly elevated, leading to the theory of multistage carcinogenesis (Armitage and Doll 1954). In many cases, it is a sequence of events, acquired somatic mutations, or simply polygenic components with strong epigenetic effects, such as in the case of brain tumours (Suter et al. 2020).

**Fig. 1:**
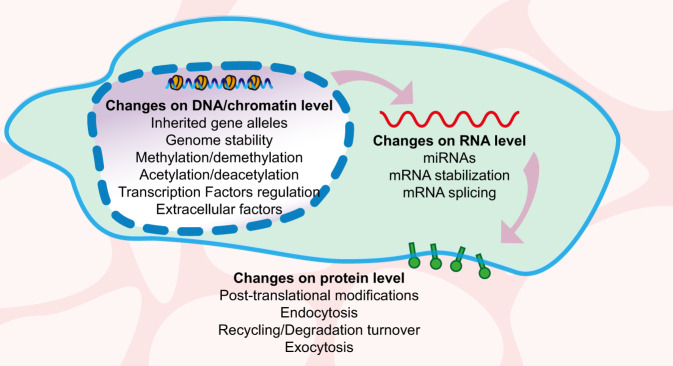
Overview of factors contributing to the expression of immune checkpoint proteins. The illustration summarises the main elements that change, positive- or negatively, the presentation of immune checkpoints at the membranes of both sides of the immunological synapse. This review will focus on the changes at DNA/chromatin and RNA levels.

One of the factors which determine a patient’s prognosis and possible response to immunotherapy is tumour immunogenicity—the recruitment of T cells to the tumour environment. Tumour cells express immune checkpoint genes to suppress the immune response by the host and facilitate tumour cells’ survival. The expression of these genes is the product of the complex interplay between several factors, including the organism’s genotype (in most cases Mendelian inheritance), genetic instability level, epigenetic factors (non-Mendelian inheritance) as well as immune cells of the host, to name just a few (Fig. 1). Here we will discuss the recent advances in the understanding of the key mechanisms controlling the expression of checkpoint genes on tumour cells.

## Overview of the immune checkpoint genes regulation in cancer

**Fig. 2:**
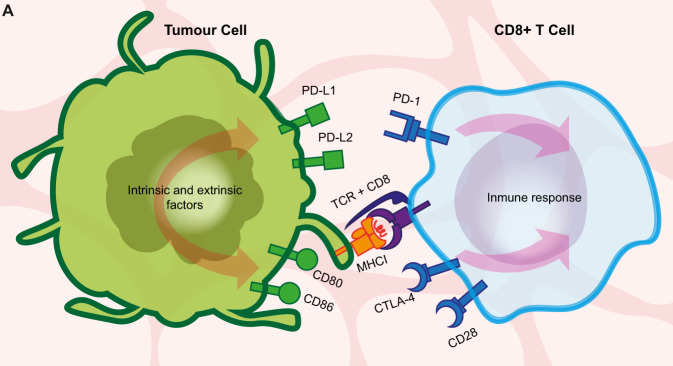
Overview of the mechanism of immune evasion at the immunological synapse of CD8+ T lymphocytes. The cartoon represents a simplified diagram of an immunological synapse between a tumour cell (left) and CD8+ T lymphocyte (right), depicting the direct interactome of two best-studied immune checkpoint proteins of clinical relevance, Programmed Cell Death Protein 1 (PD-1) and Cytotoxic T-lymphocyte-associated protein 4 (CTLA-4, see main text). Antigens as presented by the MHCI at the surface of a tumour cell (left) are recognised by CD8+ T lymphocytes (right) with the binding of the TCR receptor in association with the CD8 protein. Immune checkpoint proteins suppress the activation of the immune response upon recognition by the CD8+ T cell of additional proteins in the target cell membrane. They include PD-L1 and PD-L2, both recognised by PD-1; and CD80 and CD80, which bind to both CTLA-4 and CD28 at the T cell. A series of factors, including the host genotype, genome instability of the developing tumour and epigenetic mechanisms, change the profile of presentation of these and other proteins.

One of the most incredible powers of the human immune system is its constant ability to recognise cells as being ‘foreign’ or ‘self’, thus, to distinguish perfectly between self-antigens and non-self-antigens. Within this complex process, it is important to mention the immune checkpoint molecules: small proteins present at the cell’s membrane (Fig. 2). They need to be activated or deactivated to trigger an immune response or, even more crucial, to prevent the immune system from attacking.

T cell-dependent immune mechanisms are known to protect an organism from cancer (Dustin 2014). The details of this mechanism are still to be discovered, however, it is clear that there must be a hierarchy of molecules, receptors and their ligands, inside the ~15 nm gap formed between the T cell and an antigen-presenting cell (either APC or cancer cell) (Grakoui et al. 1999; Dustin and Colman 2002; Brossard et al. 2005; Dustin 2014). This gap with all its molecules and yet-to-be-discovered complex interactions is termed an immunological synapse. The concept is known from the 1960s or maybe earlier, however, the term was coined around 1984 for its similarity with the synapse of the nervous system (Norcross 1984; Yokosuka and Saito 2010). It has been defined as a unique molecular architecture involved in recognition and signalling, with adhesion molecules, receptors and ligands being structurally and kinetically organised in order to activate/inhibit T cell reaction (Monks et al. 1998; Grakoui et al. 1999; Yokosuka and Saito 2010). At first, the concept was bound to the interface between the T cell and APC, but with the influx of knowledge and our understanding of its complexity, it was expanded around the early 2000s to include any interface formed by the immune cells on one side, and another immune cell on the other, or between immune cell and any host target cell (Fleire et al. 2006; McCarthy et al. 2007; Orange 2008; Yokosuka and Saito 2010).

Even though checkpoint proteins are absolutely necessary for the proper functioning of the immune system, in some situations checkpoint molecules might help the disease to progress. This is the scenario we often observe in cancer—tumour cells at some point of cancer development might escape from immune recognition and attack. One of the well-described mechanisms used by cancer cells to escape from immune system recognition is simply to stop the immune cells (mostly lymphocytes) from launching an attack on them (Yokosuka and Saito 2010; Snyder 2016). Now it is clear that many cancer cells use checkpoint molecules to be recognised as ‘self’ and therefore avoid being attacked by the human immune system (Pardoll 2012). This process is known today as immune evasion and is extremely important in cancer management, including recurrent and metastatic disease (Pardoll 2012; He et al. 2015; Spranger 2016; van Elsas et al. 2020). Therefore, it is not surprising that drugs that target checkpoint proteins and reverse their actions are of great promise in oncology (Pardoll 2012; He et al. 2015; Zhang et al. 2021).

The very first checkpoint protein discovered was Programmed Cell Death Protein 1, PD-1, which has been investigated since the 1990s (Fig. 2). It functions as an off-switch, keeping T cells from launching an attack (Snyder 2016; Nguyen and Dobosz 2017; Gopalakrishnan et al. 2018; Ishida 2020; Patsoukis et al. 2020). To date, two ligands of PD-1 molecules have been discovered: PD-L1 and PD-L2. The PD-1/PD-L1 interplay has been much better investigated than the PD-1/PD-L2 axis, which has resulted in several PD-L1 inhibitors being approved by the FDA already (Dong et al. 2017; Ishida 2020; Zhang et al. 2021). Both ligands are expressed mostly on antigen-presenting cells but also frequently on cancer cells to protect them from being attacked (Dong et al. 2017).

Cytotoxic T-lymphocyte-associated protein 4 (CTLA-4) is another major checkpoint protein that limits T cell activity (Fig. 2 and Bashyam 2007). Its two ligands, CD80 and CD86, also bind the costimulatory receptor CD28, although with a lower affinity. Hence, at low expression levels as observed in cancer cells, these two receptors are inhibitory and protect cancer cells from being attacked (Li et al. 1996; Collins et al. 2002; Tirapu et al. 2006).

Despite the overall similarity in their effects on T cells, CTLA-4 and PD-1 operate at distinct stages during immune cell activation, with CTLA-4 required earlier than PD-1 (Fife and Bluestone 2008). Due to this difference in their action, it is not surprising that combinatorial treatments with inhibitors of both checkpoint proteins are proved to have higher efficacy than monotherapies, although their potential adverse effects might be more severe or even life-threatening (Rotte 2019). Using PD-1/PD-L1 and/or CTLA-4 inhibitors results in much longer overall survival rates, especially among melanoma, lung and bladder cancer patients (Hodi et al. 2010; Schachter et al. 2017; Naik et al. 2021).

It is worth mentioning that checkpoint inhibitors are also known for their side effects. They are usually transient, however, in some cases, they are permanent, and more severe for the CTLA-4 axis than PD-1/PD-L1 or any other known drug targeting immune checkpoint molecule in clinical trials (Brahmer et al. 2012; Khoja et al. 2017; reviewed nicely in Spiers et al. 2019). Despite the huge success of checkpoint inhibitors in immunotherapy, many patients treated with immunotherapeutic agents experience immune-related adverse events. These are mostly in the form of clinical autoimmunity, which may range from mild to severe or even life-threatening, impacting successful cancer treatment (Burke et al. 2021). Autoimmunity, preferably benign autoimmunity, is exactly what needs to be achieved in most cases, however, the degree of impact, as well as the strength and direction of the response, should be strictly controlled, if possible (Cohen 2014). The strongest and most frequent autoimmune reactions are observed in the case of combinatorial therapies when two drugs are administered together, but the onset of immune toxicity is variable: skin toxicities often manifest early, followed by pulmonary or gastrointestinal manifestations slightly later. Colitis, hepatitis, or endocrinopathies appear relatively late and last longer (Amos et al. 2011; Brahmer et al. 2018; Burke et al. 2021). It is important to note that, however dangerous, treatment-related immunotoxicity has been linked with better tumour response to the administered immunotherapy (Amos et al. 2011).

Additionally, some inherited susceptibilities, including HLA haplotypes or single-nucleotide polymorphisms (SNPs) in HLA genes, are usually highly predictive of the risk of developing specific autoimmune syndromes and, thus, may contribute to the development of immune checkpoint inhibitor-induced autoimmunity (Theofilopoulos et al. 2017; Marchand et al. 2019). Therefore, we hypothesise that patients developing severe autoimmunity in response to immunotherapy may have some underlying genetic predispositions. Altogether, the development of novel, less toxic combinatorial therapies and stratification of patients by predicted responses and adverse reactions require understanding the expression, regulation and interactions of checkpoint genes alongside their modulation by host and tumour genotypes.

To date, a broad repertoire of checkpoint genes has been identified, with at least 22 of them being expressed at the tumour side of the immunological synapse (Dobosz et al. 2020). On the one hand, this opens an opportunity for a versatile spectrum of combinatorial treatments for personalised immunotherapy (Qin et al. 2019). The expression of checkpoint genes in the tumour cell membrane is a product of polygenic inheritance and epigenetic regulation (Fig. 1 and Table 1), which are discussed below.

**Table 1:** List of factors regulating the immune checkpoints PD-1 and CTLA-4.

**Transcription factors and signalling pathways controlling the expression of checkpoint genes**		
Gene symbol	References	
*BACH2*	(Roychoudhuri et al. 2013, 2016; Afzali et al. 2017; Dobosz et al. 2020)	
*MAFK*	(Toki et al. 1997; Dobosz et al. 2020)	
*NFE2L2*	(Dobosz et al. 2020)	
*CTCF*	(Oreskovic et al. 2022)	
*TCF1*	(Li et al. 2021)	
Interferon type II IFN-γ	(Zerdes et al. 2018)	
Epidermal growth factor receptor (EGFR)	(Ciardiello and Tortora 2008; Akbay et al. 2013)	
**miRNAs controlling the expression of checkpoint genes**		
miRNA	Target gene	References
miR-200, miR-34a, miR-15, miR-16, miR-17-5p, miR-33a, miR-138-5p, miR-140, miR-142, miR-152, miR-155, miR-193a-3p, miR-195, miR-324-5p, miR-338-5p, miR-340, miR-383, miR-424, miR-497-5p and miR-513	*PD-L1*	(Chen et al. 2014; Wang et al. 2015; Wang and Wang 2017; Danbaran et al. 2020; Skafi et al. 2020)
miR-9, miR-105, 487a-3p	*CTLA-4*	(Houshmand et al. 2012; Jebbawi et al. 2014; He et al. 2018; Zurawek et al. 2018)
miR-18a	*SOX6* (regulator of PD-L1)	(Dong et al. 2018)
miR-15a, miR-16, miR-24, miR-95, miR-126, miR-210,	*FOXP3* (regulator of CTLA-4)	(Skafi et al. 2020)
**Epigenetic regulators of checkpoint genes with a known role in cancer**		
Protein(s)	References	
DNMT3A, DNMT3B, DNMT1	(el-Deiry et al. 1991; Patra et al. 2002; Girault et al. 2003; Lin et al. 2010; Zhang et al. 2020)	
TET1-3	(Rasmussen and Helin 2016)	
Polycomb Repressive Complex 2 (PRC2)	(Béguelin et al. 2013; Qamra et al. 2017; Zingg et al. 2017; Gan et al. 2018; Kim et al. 2020)	
HDACs	(Minucci and Pelicci 2006; Woods et al. 2015; Booth et al. 2017; Banik et al. 2019; Li et al. 2021)	

## Regulation of immune response-related genes by transcription factors and host genetics

Transcription factors are molecules that control the rate of transcription through binding to the gene promoter’s specific sequence (Lee and Young 2000; Lambert et al. 2018). They often work by forming bigger complexes, composed of many subunits, but in some cases, they act alone. Their main mechanism of action is to promote or repress the recruitment of RNA polymerase, therefore inducing or inhibiting the transcription process of a certain gene (Latchman 1997; Lambert et al. 2018). Each transcription factor has a DNA-binding domain that allows it to bind to very specific DNA sequences, usually located inside the promoter region of a gene (Latchman 1997; Lee and Young 2000; Lambert et al. 2018). Such specific sequences are often localised near the transcription start site, inside the promoter, or they can be localised at distant locations, including enhancer regions or inside introns (Latchman 1997).

Since the re-discovery of immunotherapy remains a relatively young area, after several decades of immunotherapy rejection (Zhang and Zhang 2020), there are many ongoing experiments in the field of regulation of the immune checkpoint genes by transcription factors. One of the first results delivered to date concerns bladder cancer. Among at least 22 checkpoint genes described so far, the expression of seven of these genes is highly correlated. High expression of two of these, HVEM and CD277, is associated with a better prognosis in bladder cancer (Dobosz et al. 2020). These findings resemble the co-expression of mRNAs for immune checkpoint proteins present at the T cell interface of the immunological synapse (Chen and Flies 2013; Nirschl and Drake 2013; Schnell et al. 2020; Waldman et al. 2020). If these results are corroborated at the protein level, it may prove that co-inhibition occurs concurrently by several checkpoint proteins. From a clinical point of view this would be a clear sign that targeting a single checkpoint co-inhibitor (such as PD-L1), or even two (PD-L1 and CTLA-4), may not be enough, as observed in many cancer patients that do not respond to the immunotherapy administered.

**Fig. 3:**
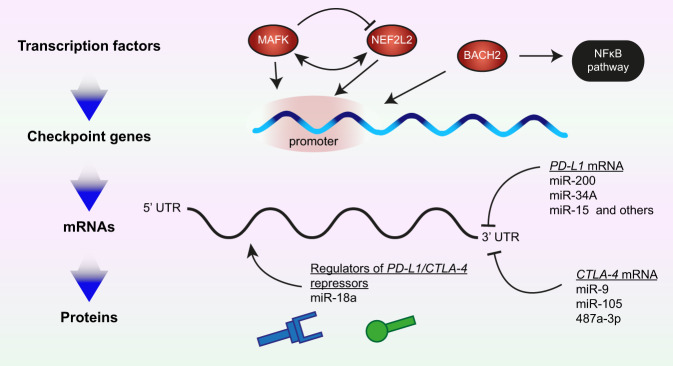
Mechanisms regulating the expression of immune checkpoint genes (1). Expression of specific immune checkpoints proteins, such as PD-L1 and CTLA-4, correlates with transcription factors (top) and microRNAs (miRNAs, bottom). Some of these interactions are direct, for example for the transcription factors MAFK, NEF2LD and BACH2; while others are indirect, for example, miR-18a targets a negative regulator of PD-L1. Some of the regulators interact with each other in both positive and negative manners (for example MAFK over NEF2L2), and others have existing functions in cellular routes closely related to cancer pathogenesis (BACH2).

Moreover, the putative binding sites of three transcription factors—BACH2, MAFK and NFE2L2—are in the promoter regions of the analysed checkpoint genes (Fig. 3 and Dobosz et al. 2020). Indeed, the BACH2 transcription factor is fundamental in several pathways in the immune system (Roychoudhuri et al. 2013, 2016). BACH2 is also involved in the NFκB signalling pathway, itself being cardinal in bladder cancer pathogenesis (Fig. 3 and Dobosz et al. 2020). Dysfunction of BACH2 is common in lymphomas (Sasaki et al. 2000), whereas *BACH2* mutations are associated with autoimmune disease, akin to those in immune checkpoint genes, and cause Mendelian monogenic primary immunodeficiency (Afzali et al. 2017).

The second transcription factor identified in the regulation of immune checkpoint genes in bladder cancer cells is MAFK (Fig. 3 and Dobosz et al. 2020). This leucine-zipper-type transcription factor is a powerful transcriptional regulator that cooperates with the NFE2 transcription factor family, hence often acting as a transcription enhancer or inhibitor, depending on the protein interactions (Toki et al. 1997; Kannan et al. 2012). Moreover, MAFK may also act as a competitive repressor of other NFE2-related transcription factors, successfully inhibiting their binding sites (Fig. 3 and Kataoka et al. 1995; Toki et al. 1997).

Considering their role in the immune response, inherited mutations in both *PD-1* and *CTLA-4* are linked with autoimmune diseases (Ogishi et al. 2021; Qi et al. 2021). Nevertheless, there are several cancer-related aberrations already described that change the expression of the immune checkpoint genes. Some of these aberrations impact transcription factors, whereas other changes impact immune checkpoint genes themselves (for changes impacting PD-L1 and CTLA-4 genes see review Zhang et al. 2021). For example, in Hodgkin’s lymphoma frequent amplifications and translocations of the 9p24.1 region, where the *PD-L1* gene sits, are known to upregulate expression of this gene (Roemer et al. 2016; Wienand et al. 2019; Zhang et al. 2021). Similar changes have been observed in other cancer types, for example, non-small cell lung carcinoma (NSCLC), small-cell lung carcinoma (SCLC), oral squamous cell carcinoma, gastric cancers (The Cancer Genome Atlas Research Network 2014; Ikeda et al. 2016; Straub et al. 2016; George et al. 2017). In contrast, another study reported no correlation between amplification of *PD-L1* gene copy number with altered immune estimates in a range of solid tumours, including melanoma and lung adenocarcinoma (Siemers et al. 2017), further highlighting the multifactorial nature of immune checkpoint gene regulation. Although this field remains relatively young, recent studies have revealed the complexity of the inherited component in the expression of checkpoint genes. *PD-L1* gene expression is lower in women than men for some cancers but not others (e.g. head and neck squamous cell carcinoma vs. mesothelioma). The expression of *PD-L1* is generally lower in individuals with predicted African ancestry, although no single *cis*-expression Quantitative Trait Locus (eQTL) correlated with *PD-L1* expression (Thorsson et al. 2018). Nevertheless, immune-related genes appear to be enriched in eQTLs and their expression is more impacted by SNPs relative to other genes (Lim et al. 2018). For example, eQTLs significantly affect the expression of Endoplasmic Reticulum Aminopeptidase 2 (ERAP2). Its low expression levels are associated with better overall survival in patients with the luminal subtype of bladder cancer in the anti-PD-L1 (atezolizumab) urothelial bladder cancer phase 2 clinical trial (Lim et al. 2018). This association, however, may be at least in part due to an indirect effect of the induction of ERAP2 expression by a widely known proinflammatory cytokine, interferon type II IFN-γ (Tanioka et al. 2003). Curiously, in cancer setting IFN-γ also promotes PD-L1 expression, although this is prone to further regulation that affects entire immune signalling pathways involving many cytokines production and activity (reviewed in Zerdes et al. 2018). A recent study confirmed that the IFN-γ pathway is the most significant regulator of basal and inducible PD-L1 expression using a CRISPR-Cas9 genetic screen with a sgRNA library (Oreskovic et al. 2022). At the same time, *CTCF*, which encodes a transcription factor and is the key regulator of three-dimensional chromatin organisation, was identified as a strong suppressor gene of PD-L1 in this screen. This example bridges genetic and epigenetic mechanisms of immune checkpoint gene regulation, with the latter being discussed in-depth in the following sections.

Finally, accumulating evidence suggests that several signalling pathways involved in oncogenesis, such as pathways regulating tumour cell proliferation and progression, may also promote checkpoint gene expression. This is especially well described for *PD-L1* expression: for example, epidermal growth factor receptor (EGFR) mutations in NSCLC epithelial cells have been shown to induce and enhance *PD-L1* expression (Ciardiello and Tortora 2008). On the other hand, EGFR inhibitors used in NSCLC patient treatment significantly lowered the expression of *PD-L1* (Ciardiello and Tortora 2008; Akbay et al. 2013). Such observations suggest that EGFR signalling itself, in certain conditions, may cause the immune escape of tumour cells.

## Epigenetic regulation of immune response

### miRNA

MicroRNAs (miRNAs, miRs) are abundant small non-coding RNAs composed of 22–24 nucleotides (Hornstein and Shomron 2006). They play an important role in post-transcriptional gene suppression and have been reported to be involved in multiple cellular processes, such as differentiation, morphogenesis and tumorigenesis (Kloosterman and Plasterk 2006; Aqeilan et al. 2010; Zhao et al. 2017). miRNAs usually target the 3′untranslated region (UTR) and less frequently the 5′UTR or the coding sequence of their target mRNA (O’Brien et al. 2018). Each miRNA molecule targets tens to hundreds of mRNAs (Filipowicz et al. 2008). Additionally, some mRNA targets are combinatorically affected by several different miRNA molecules, increasing the complexity and precision of post-transcriptional regulation, and fine-tuning the level of gene expression (Hornstein and Shomron 2006). Both loss-of-function mutations and overexpression of miRNA are found in a wide range of cancers, demonstrating that they may have roles as both oncogenes and tumour-suppressors (Kloosterman and Plasterk 2006; Zhao et al. 2017). Half of all miRNA genes are located in genomic regions known to be associated with cancer or in fragile sites often altered in human cancers (Calin et al. 2004).

Therefore, it is not surprising that the expression of miRNA was linked to that of immune checkpoint genes, regulating them both through directly binding their mRNA and indirectly through modulating other targets (Fig. 3). Thus, miR-200, a well-established regulator of epithelial-to-mesenchymal transition, directly targets PD-L1 in various cancer cells among its other targets (Chen et al. 2014). Similarly, miR-34a directly binds PD-L1 3′UTR and downregulates its expression in acute myeloid leukemia and glioma cells (Wang et al. 2015; Wang and Wang 2017). Other miRNAs that directly target PD-L1 include: miR-15, miR-16, miR-17-5p, miR-33a, miR-138-5p, miR-140, miR-142, miR-152, miR-155, miR-193a-3p, miR-195, miR-324-5p, miR-338-5p, miR-340, miR-383, miR-424, miR-497-5p and miR-513 (reviewed in Danbaran et al. 2020). In contrast, some miRNAs act indirectly; for example, miR-18a increases PD-L1 levels by targeting SOX6, which leads to activation of the WNT pathway and inactivation of p53 signalling (Dong et al. 2018). It also targets PTEN and WNK2, which contributes to PD-L1 upregulation (Dong et al. 2018).

Fewer miRNAs are known to target CTLA-4 (Fig. 3). These include miR-9, miR-105, 487a-3p (Jebbawi et al. 2014; He et al. 2018; Zurawek et al. 2018). The miR-105 is of particular interest as it can bind only the mutant variant of CTLA-4 that originates through a single-nucleotide polymorphism in the 3′UTR of CTLA-4 (He et al. 2018). This polymorphism was linked to a severe form of aggressive periodontitis (Houshmand et al. 2012), whereas miR-105 itself has a dual role in tumour progression; it is both a tumour-suppressor that inhibits tumour growth and metastasis and an oncogene that promotes tumour initiation and invasion depending on the context (Li et al. 2019). These findings highlight a complex interplay between genetically inherited alleles, epigenetic regulators, and the tumour microenvironment. Additionally, several miRNAs regulate CTLA-4 levels indirectly through targeting FOXP3 (reviewed in Skafi et al. 2020).

Recently, we performed a comprehensive bioinformatical analysis of the correlation between the expression of 21 genes known to be involved in the immunological synapse and miRNAs in bladder cancer (Dobosz et al. 2020; Stempor et al. 2021). This analysis revealed that the expression of 19 miRNAs positively correlated with checkpoint gene expression, and 27 other miRNAs negatively, indicating a high interdependency of these components in a well-connected correlation network (Stempor et al. 2021). Building on the prior indication that miRNA profiles might be more useful for cancer diagnosis and prognosis than those based on mRNA (Esquela-Kerscher and Slack 2006), combinational miRNA-mRNA profiles might therefore provide an added value for cancer diagnosis and selection of optimal strategy for immunotherapy.

### DNA methylation

**Fig. 4:**
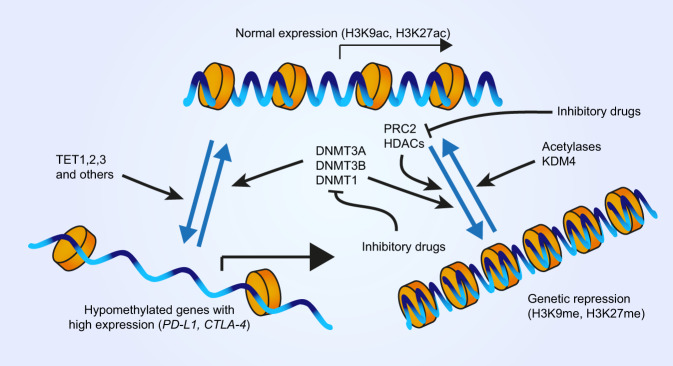
Mechanisms regulating the expression of immune checkpoint genes (2). Alongside transcription factors and miRNAs (Fig. 3), epigenetic modifications of DNA and chromatin exert a strong effect upon gene expression. Normally expressing genes in euchromatin exhibit a low degree of methylation in their associated histones, which are instead acetylated. The conversion to heterochromatin and gene silencing is preceded by deacetylation and methylation of histones (notably, the indicated Lysine residues of Histone 3, H3K). Several inhibitory drugs target the responsible enzymes and are part of combinatorial therapies. Additionally, some genes such as those involved in the immune checkpoint, have been found to exhibit hypomethylation, and therefore boosted expression, in several cancer types.

DNA methylation—the transfer of a methyl group to the C5 carbon of cytosine—of CpG-dinucleotide-rich DNA regions (‘CpG islands’) in gene promoters results in stable transcriptional silencing of gene expression (Newell-Price et al. 2000). In cancer cells, global hypomethylation of DNA is often accompanied by hypermethylation of specific genes, particularly tumour-suppressor genes, promoting cancer proliferation, invasion and survival (Esteller et al. 2001). Indeed, methylation of DNA at certain CpG islands epigenetically suppresses CTLA-4 gene expression in head and neck squamous cell carcinoma (de Vos et al. 2020). Unexpectedly, it appears to be the opposite for some other checkpoint genes on the tumour side of the immunological synapse (Fig. 4). Thus, the *PD-L1* promoter was found to be frequently hypomethylated in non-small cell lung cancers resulting in overexpression of the molecule (Kowanetz et al. 2018). A more recent systematic study of 8186 solid tumours from 30 tumour types in The Cancer Genome Atlas (TCGA) found that while the overall methylation of the immune synapse genes was most similar to the tissue of origin, the immune checkpoint genes were hypomethylated relative to the normal tissue (Berglund et al. 2020). This hypomethylation negatively correlated with gene expression and recruitment of T cells to the tumour microenvironment, giving an insight into a potential mechanism of immune response evasion by tumour cells (Berglund et al. 2020). However, it is important to mention that at least in some cases changes in the expression of immune genes preceded demethylation of CpG islands at their distal enhancers (Pacis et al. 2019), raising questions about the causality between changes in DNA methylation and expression of checkpoint genes and the mechanisms of these changes.

The mechanism of CpG island hypomethylation of immune checkpoint gene promoters is one of the major gaps in current understanding. In general, DNA methylation is established by DNA methyltransferases (DNMTs), in particular, it is placed de novo mostly by DNMT3A and DNMT3B and maintained by DNMT1 (Fig. 4 and Subramaniam et al. 2014). Overexpression of DNMTs is common in cancers including the four most common types—lung, breast, gastric and prostate (el-Deiry et al. 1991; Patra et al. 2002; Girault et al. 2003; Lin et al. 2010; Zhang et al. 2020). At least in the case of DNMT1, this might be due to the loss of repression by p53, which is lost in >50% of human cancers (Lin et al. 2010; Zhang et al. 2020). Consistently, there is Mendelian inheritance of both increased and reduced cancer risk due to particular genetic variants of all three DNMTs (Kullmann et al. 2013; Li et al. 2016). Concurrently, TET1-3 enzymes, which remove DNA methylation, are often mutated in cancers (Fig. 4 and Rasmussen and Helin 2016). Not surprisingly, DNMT inhibitors, such as Azacitidine and Decitabine, appear to be promising antitumour chemotherapy drugs (Derissen et al. 2013; Gu et al. 2021; Ma and Ge 2021). However, considering that the resulting reduction in global DNA methylation might further promote tumour evasion of the immune response while reactivating tumour-suppressor genes, it might be beneficial to administer them alongside immune checkpoint inhibitors. Indeed, clinical trials suggest the high efficacy of such combinatorial administration in various cancer types including non-small cell lung cancer and recurring ovarian cancer patients (Villanueva et al. 2020; Hu et al. 2021). However, a confident selection of such therapy might require a better understanding of the interactions between DNA methylation and checkpoint gene expression, and then a prior assessment of the methylation status of tumour cells.

### Histone modifications and the loss of heterochromatin

The genome of all organisms is organised into chromatin—a complex of DNA, RNA and associated proteins (Armstrong 2014). At the most basic level, ∼147 base pairs of DNA are wrapped around the four core histones H2A, H2B, H3, and H4 that form a nucleosome (Armstrong 2014). Multiple posttranslational modifications of the N-terminal tails of these histones are the determining factor for chromatin structure and function (Bannister and Kouzarides 2011). In particular, methylation of the histone 3 lysine 9 (H3K9me) and lysine 27 (H3K27me) are the major marks of heterochromatin—compacted and largely silenced chromosomal regions—in eukaryotes (Saksouk et al. 2015). Conversely, acetylation of these histones (H3K9ac and H3K27ac) and methylation of histone 3 lysine 4 (H3K4me) correlate with active chromatin (Roth et al. 2001; Howe et al. 2017).

Loss of heterochromatin is a hallmark of cancer, which is in part responsible for genomic instability in cancer cells and correlates with reduced global levels of H3K9me3 and H3K27me3 (Carone and Lawrence 2013; Morgan and Shilatifard 2015; Gurrion et al. 2017). Consistently, the demethylase KDM4, which removes di- and trimethylation of H3K9 (Fig. 4), is frequently overexpressed in a wide variety of cancers (Berry and Janknecht 2013; Gurrion et al. 2017). At the same time, the Polycomb Repressive Complex 2 (PRC2) places the H3K9me3 mark (Fig. 4). Overexpression, gain-of-function and loss-of-function mutations of the PRC2 catalytic subunit, the highly conserved Enhancer of zeste homolog 2 (EZH2) histone methyltransferase, are also frequently observed in many cancers (Gan et al. 2018). Expression of PRC2 correlates with T cell infiltration of melanomas and gastric adenocarcinomas, at least in part by promoting the usage of alternative promoters and, thus, leading to reduced levels of N-terminal immunogenic peptides and tumour immunoreactivity (Qamra et al. 2017; Zingg et al. 2017). This alone is sufficient to suggest a potential therapeutic benefit of using PRC2 inhibitors, such as GSK503 (Béguelin et al. 2013), alongside immune checkpoint inhibitors.

Furthermore, PRC2 appears to contribute to the development of resistance to anti-CTLA-4 immunotherapy. In mouse melanoma models, immunotherapy with anti-CTLA-4 antibodies leads to increased expression of PRC2 components including EZH2 and upregulation of PD-L1, which together help tumour cells to evade the immune response despite the treatment (Zingg et al. 2017). Inhibition of EZH2 by either siRNA or drug treatment reduced the size of skin melanomas and increased survival of the mice, but also reduced PD-L1 mRNA levels (Zingg et al. 2017). While results of clinical trials are yet to come, EZH2 inhibitors may revolutionise combinatorial immunotherapy, in particular, in cancers that normally have poor immunogenicity (Kim et al. 2020). However, this also demonstrates the complexity of interactions between checkpoint inhibitors and epigenetic machinery, whereby modulating epigenetic factors therapeutically may not always result in the predicted change in gene regulation.

An alternative approach to targeting histone methylation is to manipulate the mutually exclusive histone acetylation, which promotes open chromatin (Armstrong 2014). It has been shown that histone deacetylase (HDAC) domain mutations in several transcription factors, including TCF1, promote the expression of CTLA-4 in some T helper cell subsets, such as T follicular helper cells (Li et al. 2021). Although some HDACs are overexpressed in cancer cells, there is no apparent causative relationship between this overexpression and oncogenesis (Banik et al. 2019). However, inhibitors of HDACs are long known to slow down cancer progression by inducing cell cycle arrest, cell death and differentiation in tumour cells (Minucci and Pelicci 2006). Recently, emerging evidence indicates that they also modulate the immunological synapse (Fig. 4). In particular, they increase PD-L1 expression in several cancer models including hepatocellular carcinoma, anaplastic thyroid cancer and melanoma (Woods et al. 2015; Llopiz et al. 2019; Hegedűs et al. 2020). Therefore, a monotherapy with HDAC inhibitors alone may promote evasion of the immune response by cancer cells, limiting the beneficial effects of HDAC inhibitors. At the same time, upregulation of PD-L1 expression may potentiate the cancer response to anti-PD-L1 immunotherapy. Indeed, several recent studies demonstrate a striking potential of combining HDAC inhibitors with anti-PD-L1, reducing tumour growth and survival in melanoma, breast cancer, lung cancer and hepatocellular carcinoma models (Woods et al. 2015; Booth et al. 2017; Llopiz et al. 2019; Li et al. 2021). Moreover, the pan-HDAC inhibitor Belinostat improves the efficacy of anti-CTLA-4 therapy in the hepatocellular carcinoma model (Llopiz et al. 2019). This could be due to elevated expression of CD86 as was observed following treatment with a variety of HDAC inhibitors in acute myeloid leukaemia cells (Maeda et al. 2000). The triple therapy with Belinostat, anti-CTLA-4 and anti-PD-L1 resulted in complete tumour rejection in the hepatocellular carcinoma model (Llopiz et al. 2019). Similarly, pan-HDAC inhibitors AR42 or sodium valproate enhanced the efficacy of both anti-CTLA-4 and anti-PD-L1 therapies in melanoma models (Booth et al. 2017). At the same time, multiple HDACs appear to have overlapping functions in the regulation of PD-L1, which makes inhibitors of specific HDACs less attractive for combinatorial therapies (Booth et al. 2017). Such redundancies highlight the complexity of epigenetic regulation and underscore the importance of further studies for understanding the effects of individual HDAC inhibitors alone and in combination with immunotherapies.

## Checkpoint inhibitor genes as biomarkers for cancer clinical trials, companion diagnostics and drug discovery

Despite its promise, the current success rates of cancer immunotherapy are low—based on 6 approved checkpoint inhibitor drugs in 2018, the estimated percentage of responders is 12.46% (Haslam and Prasad 2019). The majority of patients do not respond to treatment and others suffer from adverse effects (Ottaviano et al. 2019). As levels of checkpoint gene expression have good predictive power for a patient’s survival chance (Havel et al. 2019), the checkpoint proteins are promising biomarkers for companion diagnostics—a test that indicates if a particular drug is viable for a patient (Arora et al. 2019). Immunostaining of a single protein of interest—such as PD-L1—can be used to assess the target activation on the tumour cells and determine if the concentration of target protein is high enough for a drug to work (Akhtar et al. 2021). This in turn can contribute to improved clinical outcomes and reduce the cost of new cancer treatments for healthcare systems (Akhmetov et al. 2015).

In clinical practice, the immunohistochemistry (IHC) measurements of PD-L1 are routinely used to stratify patient cohorts for clinical trials. However, setting up a threshold even for a single protein is a challenging problem that can determine the success or failure of a clinical trial. For example, two clinical trials of checkpoint inhibitor drugs for Non-Small Cell Lung Cancer: KEYNOTE-024 (Keytruda) (Reck et al. 2016 and https://clinicaltrials.gov/ct2/show/NCT02142738) by Merck and CheckMate-026 (Opdivo) (Carbone et al. 2017 and https://clinicaltrials.gov/ct2/show/NCT02041533) by Bristol-Myers used IHC to determine the levels of PD-L1 biomarker. The major difference in the design between these trials was the biomarker threshold level—set more restrictively in KEYNOTE-024 (Keytruda). Clinical trials for Keytruda met their endpoint successfully with 305 enroled participants, while Opdivo was unsuccessful with a cohort of 1325 participants (Reck et al. 2016; Carbone et al. 2017).

Lack of standardisation and reproducibility is hindering the adoption of IHC methods as companion diagnostics and precision medicine tools outside of clinical trials (Ilie and Hofman 2017). For example, more than four different assays and antibodies are used for PD-L1, each developed aside an immune checkpoint inhibitor. Using next-generation RNA sequencing (RNA-seq) instead of IHC might help standardise the assays and improve reproducibility (Conroy et al. 2019). However, there is still the problem of selecting the right biomarkers and setting a protein concentration or expression threshold at the optimal level. The complexity of the regulatory network in the immunological synapse described above makes a universal set of biomarkers that would work across a wide set of drugs not feasible (Disis 2010).

Even for well-established and clinically proven biomarkers, such as the level of PD-L1 in cancer cells, the final therapeutic outcome might be influenced by expression levels of genes (and the resulting protein levels) upstream or downstream in the regulatory pathway. Hence, a panel of multiple biomarkers may produce better prediction power than a single biomarker. Indeed, adding the RNA-seq expression estimates of other biomarkers increases the predictive power of anti-PD-1 therapy efficacy in comparison to using PD-L1 levels alone (Ayers et al. 2017; Prat et al. 2017; Morrison et al. 2018)

IHC is also challenging technically for testing multiple biomarkers (Tan et al. 2020). In contrast, technologies such as multiplexed proteomic (e.g. Olink (Petrera et al. 2021)) allow measuring the levels of hundreds of proteins, whereas RNA-seq allows establishing these numbers by proxy (by measuring gene expression levels, but not the actual levels of proteins) genome-wide (Conroy et al. 2019). In addition to biomarker selection problems (Spencer et al. 2016; Bai et al. 2020), the complexity of building panels of multiple biomarkers involves setting up thresholds for a combination of factors (Havel et al. 2019; Sajjadi et al. 2020). Theoretically adding more factors should increase predictive power, but it also requires more complex calculations and data to set up the thresholds at the right levels. This can be facilitated using machine learning data-driven approaches (Bradley and Cannings 2022) that can learn parameters from the data, namely, to set up the thresholds at optimal (given the limitations of the data) levels (Leclercq et al. 2019). This usually requires collecting a big set of patient data (~millions of samples)—both clinical histories and biochemical measurements for the biomarker panel (Swan et al. 2015), which, at present, is prohibitive to generating such models (Krassowski et al. 2020). However, recent developments in machine learning, for example, utilising a Bayesian interface (Polson and Sokolov 2017), mean that it is possible to train the models with datasets that are an order of magnitude smaller (Assawamakin et al. 2013; Zhang and Ling 2018; Dockès et al. 2021; Ko et al. 2021).

Furthermore, such models could be augmented by ex-vivo data that are much faster and cheaper to generate; for example, ex-vivo drug library screens could be used for initial training, while actual patient data would be used for polishing and validation of the predictive model (Clark 2009; Makvandi et al. 2016). Enriching protein level data with other multi-omics data—genomic variants, methylation, histone modifications and metabolomics data is expected to yield even better predictive power for precision medicine (Olivier et al. 2019). Integrating multiple modalities of omics data can help us not only understand correlative associations between protein levels and clinical outcomes but also learn the underlying biological processes and determine causative associations (Qin et al. 2019; Subramanian et al. 2020). In the future, such methods will allow us to go further than simple companion diagnostics and enable the selection of truly personalised drug combinations for cancer patients (John et al. 2020).

Summing up, using checkpoint inhibitor genes as biomarkers already yields clinical results. In combination with immunotherapy drugs, such biomarkers show the potential to be a real game-changer for cancer patients. In our opinion, we will see more high precision diagnostic tools based on multi-factor panels and utilising machine learning methods for inference of clinical outcomes. Adding multi-omics data will increase both prediction power and the number of applications of these tools.

## Conclusions/summary

Cancer immunotherapy remains an intriguing and rapidly developing field, from bench to bedside. Checkpoint inhibitor immunotherapies, despite being in their infancy, have already shown remarkable clinical effectiveness among many different cancer types. Of at least 22 known checkpoint molecules only PD-1 and CTLA-4 have been studied well enough in order to develop therapies and test them in clinical settings. With many more molecules waiting to be investigated, this field is already a great example of how connecting biomarkers discovered using classical genetic approaches such as knock out models with knowledge of epigenetic mechanisms derived from omics techniques (DNA methylation, histone modifications, small RNAs) benefits patients and further expands our knowledge about cancer.

## References

[CR1] Afzali B, Grönholm J, Vandrovcova J, O’Brien C, Sun H-W, Vanderleyden I (2017). BACH2 immunodeficiency illustrates an association between super-enhancers and haploinsufficiency. Nat Immunol.

[CR2] Akbay EA, Koyama S, Carretero J, Altabef A, Tchaicha JH, Christensen CL (2013). Activation of the PD-1 pathway contributes to immune escape in EGFR-driven lung tumors. Cancer Discov.

[CR3] Akhmetov I, Ramaswamy R, Akhmetov I, Thimmaraju P (2015). Market access advancements and challenges in “Drug-Companion Diagnostic Test” co-development in Europe. JPM.

[CR4] Akhtar M, Rashid S, Al-Bozom IA (2021). PD−L1 immunostaining: what pathologists need to know. Diagn Pathol.

[CR5] Amos SM, Duong CPM, Westwood JA, Ritchie DS, Junghans RP, Darcy PK (2011). Autoimmunity associated with immunotherapy of cancer. Blood.

[CR6] Aqeilan RI, Calin GA, Croce CM (2010). miR-15a and miR-16-1 in cancer: discovery, function and future perspectives. Cell Death Differ.

[CR7] Armitage P, Doll R (1954). The age distribution of cancer and a multi-stage theory of carcinogenesis. Br J Cancer.

[CR8] Armstrong L (2014). Epigenetics..

[CR9] Arora S, Velichinskii R, Lesh RW, Ali U, Kubiak M, Bansal P (2019). Existing and emerging biomarkers for immune checkpoint immunotherapy in solid tumors. Adv Ther.

[CR10] Assawamakin A, Prueksaaroon S, Kulawonganunchai S, Shaw PJ, Varavithya V, Ruangrajitpakorn T (2013). Biomarker selection and classification of “Omics” data using a two-step Bayes classification framework. BioMed Res Int.

[CR11] Ayers M, Lunceford J, Nebozhyn M, Murphy E, Loboda A, Kaufman DR (2017). IFN-γ-related mRNA profile predicts clinical response to PD-1 blockade. J Clin Investig.

[CR12] Bai R, Lv Z, Xu D, Cui J (2020). Predictive biomarkers for cancer immunotherapy with immune checkpoint inhibitors. Biomark Res.

[CR13] Banik D, Moufarrij S, Villagra A (2019). Immunoepigenetics combination therapies: an overview of the role of HDACs in cancer immunotherapy. IJMS.

[CR14] Bannister AJ, Kouzarides T (2011). Regulation of chromatin by histone modifications. Cell Res.

[CR15] Bashyam H (2007). CTLA-4: from conflict to clinic. J Exp Med.

[CR16] Béguelin W, Popovic R, Teater M, Jiang Y, Bunting KL, Rosen M (2013). EZH2 is required for germinal center formation and somatic EZH2 mutations promote lymphoid transformation. Cancer Cell.

[CR17] Berglund A, Mills M, Putney RM, Hamaidi I, Mulé J, Kim S (2020). Methylation of immune synapse genes modulates tumor immunogenicity. J Clin Investig.

[CR18] Berry WL, Janknecht R (2013). KDM4/JMJD2 histone demethylases: epigenetic regulators in cancer cells. Cancer Res.

[CR19] Booth L, Roberts JL, Poklepovic A, Kirkwood J, Dent P (2017). HDAC inhibitors enhance the immunotherapy response of melanoma cells. Oncotarget.

[CR20] Bradley JR, Cannings TI (2022). Data-driven design of targeted gene panels for estimating immunotherapy biomarkers. Commun Biol.

[CR21] Brahmer JR, Lacchetti C, Schneider BJ, Atkins MB, Brassil KJ, Caterino JM (2018). Management of immune-related adverse events in patients treated with immune checkpoint inhibitor therapy: American Society of Clinical Oncology Clinical Practice Guideline. JCO.

[CR22] Brahmer JR, Tykodi SS, Chow LQM, Hwu W-J, Topalian SL, Hwu P (2012). Safety and activity of anti–PD-L1 antibody in patients with advanced cancer. N Engl J Med.

[CR23] Brossard C, Feuillet V, Schmitt A, Randriamampita C, Romao M, Raposo G (2005). Multifocal structure of the T cell - dendritic cell synapse. Eur J Immunol.

[CR24] Burke KP, Grebinoski S, Sharpe AH, Vignali DAA (2021). Understanding adverse events of immunotherapy: a mechanistic perspective. J Exp Med.

[CR25] Calin GA, Sevignani C, Dumitru CD, Hyslop T, Noch E, Yendamuri S (2004). Human microRNA genes are frequently located at fragile sites and genomic regions involved in cancers. Proc Natl Acad Sci.

[CR26] Carbone DP, Reck M, Paz-Ares L, Creelan B, Horn L, Steins M (2017). First-line nivolumab in stage IV or recurrent non–small-cell lung cancer. N Engl J Med.

[CR27] Carone DM, Lawrence JB (2013). Heterochromatin Instability in cancer: from the Barr body to satellites and the nuclear periphery. Semin Cancer Biol.

[CR28] Chen L, Flies DB (2013). Molecular mechanisms of T cell co-stimulation and co-inhibition. Nat Rev Immunol.

[CR29] Chen L, Gibbons DL, Goswami S, Cortez MA, Ahn Y-H, Byers LA (2014). Metastasis is regulated via microRNA-200/ZEB1 axis control of tumour cell PD-L1 expression and intratumoral immunosuppression. Nat Commun.

[CR30] Ciardiello F, Tortora G (2008). EGFR antagonists in cancer treatment. N. Engl J Med.

[CR31] Clark DP (2009). Ex vivo biomarkers: functional tools to guide targeted drug development and therapy. Expert Rev Mol Diagnostics.

[CR32] Cohen IR (2014). Activation of benign autoimmunity as both tumor and autoimmune disease immunotherapy: a comprehensive review. J Autoimmun.

[CR33] Collins AV, Brodie DW, Gilbert RJC, Iaboni A, Manso-Sancho R, Walse B (2002). The interaction properties of costimulatory molecules revisited. Immunity.

[CR34] Conroy JM, Pabla S, Nesline MK, Glenn ST, Papanicolau-Sengos A, Burgher B (2019). Next generation sequencing of PD-L1 for predicting response to immune checkpoint inhibitors. j Immunother cancer.

[CR35] Danbaran GR, Aslani S, Sharafkandi N, Hemmatzadeh M, Hosseinzadeh R, Azizi G (2020). How microRNAs affect the PD-L1 and its synthetic pathway in cancer. Int Immunopharmacol.

[CR37] Derissen EJB, Beijnen JH, Schellens JHM (2013). Concise drug review: azacitidine and decitabine. Oncologist.

[CR178] de Vos L, Grünwald I, Bawden EG, Dietrich J, Scheckenbach K, Wiek C et al. (2020) The landscape of CD28, CD80, CD86, CTLA4, and ICOS DNA methylation in head and neck squamous cell carcinomas. Epigenetics 15:1195–1212 10.1080/15592294.2020.1754675 PMC7595594 32281488

[CR38] Disis ML (2010). Immune regulation of cancer. JCO.

[CR39] Dobosz P, Stempor PA, Roszik J, Herman A, Layani A, Berger R (2020). Checkpoint genes at the cancer side of the immunological synapse in bladder cancer. Transl Oncol.

[CR40] Dockès J, Varoquaux G, Poline J-B (2021). Preventing dataset shift from breaking machine-learning biomarkers. GigaScience.

[CR41] Dong Y, Sun Q, Zhang X (2017). PD-1 and its ligands are important immune checkpoints in cancer. Oncotarget.

[CR42] Dong P, Xiong Y, Yu J, Chen L, Tao T, Yi S (2018). Control of PD-L1 expression by miR-140/142/340/383 and oncogenic activation of the OCT4–miR-18a pathway in cervical cancer. Oncogene.

[CR43] Dustin ML (2014). The immunological synapse. Cancer Immunol Res.

[CR44] Dustin ML, Colman DR (2002). Neural and immunological synaptic relations. Science.

[CR36] el-Deiry WS, Nelkin BD, Celano P, Yen RW, Falco JP, Hamilton SR (1991). High expression of the DNA methyltransferase gene characterizes human neoplastic cells and progression stages of colon cancer. Proc Natl Acad Sci.

[CR46] Esquela-Kerscher A, Slack FJ (2006). Oncomirs—microRNAs with a role in cancer. Nat Rev Cancer.

[CR47] Esteller M, Corn PG, Baylin SB, Herman JG (2001). A gene hypermethylation profile of human cancer. Cancer Res.

[CR48] Fife BT, Bluestone JA (2008). Control of peripheral T-cell tolerance and autoimmunity via the CTLA-4 and PD-1 pathways. Immunological Rev.

[CR49] Filipowicz W, Bhattacharyya SN, Sonenberg N (2008). Mechanisms of post-transcriptional regulation by microRNAs: are the answers in sight?. Nat Rev Genet.

[CR50] Fleire SJ, Goldman JP, Carrasco YR, Weber M, Bray D, Batista FD (2006). B cell ligand discrimination through a spreading and contraction response. Science.

[CR51] Gan L, Yang Y, Li Q, Feng Y, Liu T, Guo W (2018). Epigenetic regulation of cancer progression by EZH2: from biological insights to therapeutic potential. Biomark Res.

[CR52] George J, Saito M, Tsuta K, Iwakawa R, Shiraishi K, Scheel AH (2017). Genomic amplification of CD274 (PD-L1) in small-cell lung cancer. Clin Cancer Res.

[CR53] Girault I, Tozlu S, Lidereau R, Bièche I (2003). Expression analysis of DNA methyltransferases 1, 3A, and 3B in sporadic breast carcinomas. Clin Cancer Res.

[CR54] Gopalakrishnan V, Spencer CN, Nezi L, Reuben A, Andrews MC, Karpinets TV (2018). Gut microbiome modulates response to anti-PD-1 immunotherapy in melanoma patients. Science.

[CR55] Grakoui A, Bromley SK, Sumen C, Davis MM, Shaw AS, Allen PM (1999). The immunological synapse: a molecular machine controlling T cell activation. Science.

[CR56] Gu X, Tohme R, Tomlinson B, Sakre N, Hasipek M, Durkin L (2021). Decitabine- and 5-azacytidine resistance emerges from adaptive responses of the pyrimidine metabolism network. Leukemia.

[CR57] Gurrion C, Uriostegui M, Zurita M (2017). Heterochromatin reduction correlates with the increase of the KDM4B and KDM6A demethylases and the expression of pericentromeric DNA during the acquisition of a transformed phenotype. J Cancer.

[CR58] Haslam A, Prasad V (2019). Estimation of the percentage of US patients with cancer who are eligible for and respond to checkpoint inhibitor immunotherapy drugs. JAMA Netw Open.

[CR59] Havel JJ, Chowell D, Chan TA (2019). The evolving landscape of biomarkers for checkpoint inhibitor immunotherapy. Nat Rev Cancer.

[CR60] He J, Hu Y, Hu M, Li B (2015). Development of PD-1/PD-L1 pathway in tumor immune microenvironment and treatment for non-small cell lung cancer. Sci Rep.

[CR61] He F, Zhou Y, Wang X, Li L, Geng Y, Wang Z (2018). Functional polymorphisms of CTLA4 associated with aggressive periodontitis in the Chinese Han population. Cell Physiol Biochem.

[CR62] Hegedűs L, Rittler D, Garay T, Stockhammer P, Kovács I, Döme B (2020). HDAC inhibition induces PD-L1 expression in a novel anaplastic thyroid cancer cell line. Pathol Oncol Res.

[CR63] Hodi FS, O’Day SJ, McDermott DF, Weber RW, Sosman JA, Haanen JB (2010). Improved survival with ipilimumab in patients with metastatic melanoma. N. Engl J Med.

[CR64] Hornstein E, Shomron N (2006). Canalization of development by microRNAs. Nat Genet.

[CR65] Houshmand B, Rafiei A, Hajilooi M (2012). Influence of cytotoxic T lymphocyte antigen-4 (CTLA-4) gene polymorphisms in periodontitis. Arch Oral Biol.

[CR66] Howe FS, Fischl H, Murray SC, Mellor J (2017). Is H3K4me3 instructive for transcription activation?. BioEssays.

[CR67] Hu C, Liu X, Zeng Y, Liu J, Wu F (2021). DNA methyltransferase inhibitors combination therapy for the treatment of solid tumor: mechanism and clinical application. Clin Epigenet.

[CR68] Ikeda S, Okamoto T, Okano S, Umemoto Y, Tagawa T, Morodomi Y et al. (2016) PD-L1 is upregulated by simultaneous amplification of the PD-L1 and JAK2 genes in non–small cell lung cancer. J Thorac Oncol 11:62–71 10.1016/j.jtho.2015.09.010 26762740

[CR70] Ilie M, Hofman P (2017). Reproducibility of PD-L1 assessment in non-small cell lung cancer—know your limits but never stop trying to exceed them. Transl Lung Cancer Res.

[CR71] Ishida Y (2020). PD-1: its discovery, involvement in cancer immunotherapy, and beyond. Cells.

[CR72] Jebbawi F, Fayyad-Kazan H, Merimi M, Lewalle P, Verougstraete J-C, Leo O (2014). A microRNA profile of human CD8+ regulatory T cells and characterization of the effects of microRNAs on Treg cell-associated genes. J Transl Med.

[CR73] John A, Qin B, Kalari KR, Wang L, Yu J (2020). Patient-specific multi-omics models and the application in personalized combination therapy. Fut Oncol 10.2217/fon-2020-0119 10.2217/fon-2020-0119 32462937

[CR74] Kannan MB, Solovieva V, Blank V (2012). The small MAF transcription factors MAFF, MAFG and MAFK: Current knowledge and perspectives. Biochim Biophys Acta.

[CR75] Kataoka K, Igarashi K, Itoh K, Fujiwara KT, Noda M, Yamamoto M (1995). Small Maf proteins heterodimerize with Fos and may act as competitive repressors of the NF-E2 transcription factor. Mol Cell Biol.

[CR76] Khoja L, Day D, Wei-Wu Chen T, Siu LL, Hansen AR (2017). Tumour- and class-specific patterns of immune-related adverse events of immune checkpoint inhibitors: a systematic review. Ann Oncol.

[CR77] Kim H-J, Cantor H, Cosmopoulos K (2020). Overcoming immune checkpoint blockade resistance via EZH2 inhibition. Trends Immunol.

[CR78] Kloosterman WP, Plasterk RHA (2006). The diverse functions of microRNAs in animal development and disease. Dev Cell.

[CR79] Ko S, Choi J, Ahn J (2021). GVES: machine learning model for identification of prognostic genes with a small dataset. Sci Rep..

[CR80] Kowanetz M, Zou W, Gettinger SN, Koeppen H, Kockx M, Schmid P et al. (2018) Differential regulation of PD-L1 expression by immune and tumor cells in NSCLC and the response to treatment with atezolizumab (anti–PD-L1). Proc Natl Acad Sci USA 115:E10119–E10126 10.1073/pnas.1802166115 PMC6205493 30297397

[CR81] Krassowski M, Das V, Sahu SK, Misra BB (2020). State of the field in multi-omics research: from computational needs to data mining and sharing. Front Genet.

[CR82] Kullmann K, Deryal M, Ong MF, Schmidt W, Mahlknecht U (2013). DNMT1 genetic polymorphisms affect breast cancer risk in the central European Caucasian population. Clin Epigenet.

[CR83] Lambert SA, Jolma A, Campitelli LF, Das PK, Yin Y, Albu M (2018). The human transcription factors. Cell.

[CR84] Latchman DS (1997). Transcription factors: an overview. Int J Biochem Cell Biol.

[CR85] Leclercq M, Vittrant B, Martin-Magniette ML, Scott Boyer MP, Perin O, Bergeron A (2019). Large-scale automatic feature selection for biomarker discovery in high-dimensional OMICs data. Front Genet.

[CR86] Lee TI, Young RA (2000). Transcription of eukaryotic protein-coding genes. Annu Rev Genet.

[CR87] Li H, Li W, Liu S, Zong S, Wang W, Ren J (2016). DNMT1, DNMT3A and DNMT3B polymorphisms associated with gastric cancer risk: a systematic review and meta-analysis. EBioMedicine.

[CR88] Li X, Su X, Liu R, Pan Y, Fang J, Cao L (2021). HDAC inhibition potentiates anti-tumor activity of macrophages and enhances anti-PD-L1-mediated tumor suppression. Oncogene.

[CR89] Li J, Yang Y, Inoue H, Mori M, Akiyoshi T (1996). The expression of costimulatory molecules CD80 and CD86 in human carcinoma cell lines: its regulation by interferon γ and interleukin-10. Cancer Immunol Immunother.

[CR90] Li J, Zhang Z, Chen F, Hu T, Peng W, Gu Q (2019). The diverse oncogenic and tumor suppressor roles of microRNA-105 in cancer. Front Oncol.

[CR91] Li F, Zhao X, Zhang Y, Shao P, Ma X, Paradee WJ (2021). T FH cells depend on Tcf1-intrinsic HDAC activity to suppress CTLA4 and guard B-cell help function. Proc Natl Acad Sci USA.

[CR92] Lim YW, Chen-Harris H, Mayba O, Lianoglou S, Wuster A, Bhangale T et al. (2018) Germline genetic polymorphisms influence tumor gene expression and immune cell infiltration. Proc Natl Acad Sci USA 115 10.1073/pnas.1804506115 PMC6294879 30463956

[CR93] Lin R-K, Wu C-Y, Chang J-W, Juan L-J, Hsu H-S, Chen C-Y (2010). Dysregulation of p53/Sp1 control leads to DNA methyltransferase-1 overexpression in lung cancer. Cancer Res.

[CR94] Llopiz D, Ruiz M, Villanueva L, Iglesias T, Silva L, Egea J (2019). Enhanced anti-tumor efficacy of checkpoint inhibitors in combination with the histone deacetylase inhibitor Belinostat in a murine hepatocellular carcinoma model. Cancer Immunol Immunother.

[CR95] Ma J, Ge Z (2021). Comparison between decitabine and azacitidine for patients with acute myeloid leukemia and higher-risk myelodysplastic syndrome: a systematic review and network meta-analysis. Front Pharm.

[CR96] Maeda T, Towatari M, Kosugi H, Saito H (2000). Up-regulation of costimulatory/adhesion molecules by histone deacetylase inhibitors in acute myeloid leukemia cells. Blood.

[CR97] Makvandi M, Xu K, Lieberman BP, Anderson R-C, Effron SS, Winters HD (2016). A radiotracer strategy to quantify PARP-1 expression in vivo provides a biomarker that can enable patient selection for PARP inhibitor therapy. Cancer Res.

[CR98] Marchand L, Disse E, Dalle S, Reffet S, Vouillarmet J, Fabien N (2019). The multifaceted nature of diabetes mellitus induced by checkpoint inhibitors. Acta Diabetol.

[CR99] McCarthy C, Shepherd D, Fleire S, Stronge VS, Koch M, Illarionov PA (2007). The length of lipids bound to human CD1d molecules modulates the affinity of NKT cell TCR and the threshold of NKT cell activation. J Exp Med.

[CR100] Minucci S, Pelicci PG (2006). Histone deacetylase inhibitors and the promise of epigenetic (and more) treatments for cancer. Nat Rev Cancer.

[CR101] Monks CRF, Freiberg BA, Kupfer H, Sciaky N, Kupfer A (1998). Three-dimensional segregation of supramolecular activation clusters in T cells. Nature.

[CR102] Morgan MA, Shilatifard A (2015). Chromatin signatures of cancer. Genes Dev.

[CR103] Morrison C, Pabla S, Conroy JM, Nesline MK, Glenn ST, Dressman D (2018). Predicting response to checkpoint inhibitors in melanoma beyond PD-L1 and mutational burden. J Immunother Cancer.

[CR104] Murthy P, Muggia F (2019) Women’s cancers: how the discovery of BRCA genes is driving current concepts of cancer biology and therapeutics. ecancer 13 10.3332/ecancer.2019.904 PMC6411414 30915162

[CR105] Naik GS, Buchbinder EI, Cohen JV, Manos MP, Johnson AEW, Bowling P (2021). Long-term overall survival and predictors in Anti–PD-1-naive melanoma patients with brain metastases treated with immune checkpoint inhibitors in the real-world setting: a multicohort study. J Immunother.

[CR106] Newell-Price J, Clark AJL, King P (2000). DNA methylation and silencing of gene expression. Trends Endocrinol Metab.

[CR107] Nguyen M, Dobosz P (2017). New frontiers in melanoma epigenetics—the more we know, the more we don’t know. Epigenomes.

[CR108] Nirschl CJ, Drake CG (2013). Molecular pathways: coexpression of immune checkpoint molecules: signaling pathways and implications for cancer immunotherapy. Clin Cancer Res.

[CR109] Norcross MA (1984). A synaptic basis for T-lymphocyte activation. Ann Immunol.

[CR110] O’Brien J, Hayder H, Zayed Y, Peng C (2018). Overview of microRNA biogenesis, mechanisms of actions, and circulation. Front Endocrinol.

[CR111] Ogishi M, Yang R, Aytekin C, Langlais D, Bourgey M, Khan T (2021). Inherited PD-1 deficiency underlies tuberculosis and autoimmunity in a child. Nat Med.

[CR112] Olivier M, Asmis R, Hawkins GA, Howard TD, Cox LA (2019). The need for multi-omics biomarker signatures in precision medicine. IJMS.

[CR113] Orange JS (2008). Formation and function of the lytic NK-cell immunological synapse. Nat Rev Immunol.

[CR114] Oreskovic E, Wheeler EC, Mengwasser KE, Fujimura E, Martin TD, Tothova Z (2022). Genetic analysis of cancer drivers reveals cohesin and CTCF as suppressors of PD-L1. Proc Natl Acad Sci USA.

[CR115] Ottaviano M, De Placido S, Ascierto PA (2019). Recent success and limitations of immune checkpoint inhibitors for cancer: a lesson from melanoma. Virchows Arch.

[CR116] Pacis A, Mailhot-Léonard F, Tailleux L, Randolph HE, Yotova V, Dumaine A (2019). Gene activation precedes DNA demethylation in response to infection in human dendritic cells. Proc Natl Acad Sci USA.

[CR117] Pardoll DM (2012). The blockade of immune checkpoints in cancer immunotherapy. Nat Rev Cancer.

[CR118] Patra SK, Patra A, Zhao H, Dahiya R (2002). DNA methyltransferase and demethylase in human prostate cancer. Mol Carcinog.

[CR119] Patsoukis N, Wang Q, Strauss L, Boussiotis VA (2020). Revisiting the PD-1 pathway. Sci Adv.

[CR120] Petrera A, von Toerne C, Behler J, Huth C, Thorand B, Hilgendorff A (2021). Multiplatform approach for plasma proteomics: complementarity of olink proximity extension assay technology to mass spectrometry-based protein profiling. J Proteome Res.

[CR121] Polson NG, Sokolov V (2017) Deep learning: a Bayesian perspective. Bayesian Anal 12

[CR122] Prat A, Navarro A, Paré L, Reguart N, Galván P, Pascual T (2017). Immune-related gene expression profiling after PD-1 blockade in non–small cell lung carcinoma, head and neck squamous cell carcinoma, and melanoma. Cancer Res.

[CR123] Qamra A, Xing M, Padmanabhan N, Kwok JJT, Zhang S, Xu C (2017). Epigenomic promoter alterations amplify gene isoform and immunogenic diversity in gastric adenocarcinoma. Cancer Disco.

[CR124] Qi Y, Zhao X, Liu X, Wang Y, Zhai Y, Zhang X (2021). Lupus susceptibility region containing CTLA4 rs17268364 functionally reduces CTLA4 expression by binding EWSR1 and correlates IFN-α signature. Arthritis Res Ther.

[CR125] Qin H, Niu T, Zhao J (2019). Identifying multi-omics causers and causal pathways for complex traits. Front Genet.

[CR126] Qin S, Xu L, Yi M, Yu S, Wu K, Luo S (2019). Novel immune checkpoint targets: moving beyond PD-1 and CTLA-4. Mol Cancer.

[CR127] Rasmussen KD, Helin K (2016). Role of TET enzymes in DNA methylation, development, and cancer. Genes Dev.

[CR128] Reck M, Rodríguez-Abreu D, Robinson AG, Hui R, Csőszi T, Fülöp A (2016). Pembrolizumab versus chemotherapy for PD-L1-positive non-small-cell lung cancer. N Engl J Med.

[CR129] Roemer MGM, Advani RH, Ligon AH, Natkunam Y, Redd RA, Homer H (2016). PD-L1 and PD-L2 genetic alterations define classical hodgkin lymphoma and predict outcome. JCO.

[CR130] Roth SY, Denu JM, Allis CD (2001). Histone acetyltransferases. Annu Rev Biochem.

[CR131] Rotte A (2019). Combination of CTLA-4 and PD-1 blockers for treatment of cancer. J Exp Clin Cancer Res.

[CR132] Roychoudhuri R, Clever D, Li P, Wakabayashi Y, Quinn KM, Klebanoff CA (2016). BACH2 regulates CD8+ T cell differentiation by controlling access of AP-1 factors to enhancers. Nat Immunol.

[CR133] Roychoudhuri R, Hirahara K, Mousavi K, Clever D, Klebanoff CA, Bonelli M (2013). BACH2 represses effector programs to stabilize Treg-mediated immune homeostasis. Nature.

[CR134] Sajjadi E, Venetis K, Scatena C, Fusco N (2020) Biomarkers for precision immunotherapy in the metastatic setting: hope or reality? ecancer 14 10.3332/ecancer.2020.1150 PMC7864694 33574895

[CR135] Saksouk N, Simboeck E, Déjardin J (2015). Constitutive heterochromatin formation and transcription in mammals. Epigenetics Chromatin.

[CR136] Sasaki S, Ito E, Toki T, Maekawa T, Kanezaki R, Umenai T (2000). Cloning and expression of human B cell-specific transcription factor BACH2 mapped to chromosome 6q15. Oncogene.

[CR137] Schachter J, Ribas A, Long GV, Arance A, Grob J-J, Mortier L (2017). Pembrolizumab versus ipilimumab for advanced melanoma: final overall survival results of a multicentre, randomised, open-label phase 3 study (KEYNOTE-006). Lancet.

[CR138] Schnell A, Bod L, Madi A, Kuchroo VK (2020). The yin and yang of co-inhibitory receptors: toward anti-tumor immunity without autoimmunity. Cell Res.

[CR139] Siemers NO, Holloway JL, Chang H, Chasalow SD, Ross-MacDonald PB, Voliva CF (2017). Genome-wide association analysis identifies genetic correlates of immune infiltrates in solid tumors (R Chammas, Ed.). PLoS ONE.

[CR140] Skafi N, Fayyad-Kazan M, Badran B (2020). Immunomodulatory role for MicroRNAs: regulation of PD-1/PD-L1 and CTLA-4 immune checkpoints expression. Gene.

[CR141] Smithers DW (1948). Family histories of 459 patients with cancer of the breast. Br J Cancer.

[CR142] Snyder M (2016) Genomics and personalized medicine: what everyone needs to know^®^, 1st edn. Oxford University Press. 10.1093/wentk/9780190234775.001.0001

[CR143] Spencer KR, Wang J, Silk AW, Ganesan S, Kaufman HL, Mehnert JM (2016) Biomarkers for immunotherapy: current developments and challenges. Am Soc Clin Oncol Educ Book 35:e493–503 10.1200/EDBK_160766 27249758

[CR144] Spiers L, Coupe N, Payne M (2019). Toxicities associated with checkpoint inhibitors—an overview. Rheumatology.

[CR145] Spranger S (2016). Mechanisms of tumor escape in the context of the T-cell-inflamed and the non-T-cell-inflamed tumor microenvironment. INTIMM.

[CR146] Stempor PA, Avni D, Leibowitz R, Sidi Y, Stępień M, Dzieciątkowski T (2021). Comprehensive analysis of correlations in the expression of miRNA genes and immune checkpoint genes in bladder cancer cells. IJMS.

[CR147] Straub M, Drecoll E, Pfarr N, Weichert W, Langer R, Hapfelmeier A (2016). CD274/PD-L1 gene amplification and PD-L1 protein expression are common events in squamous cell carcinoma of the oral cavity. Oncotarget.

[CR148] Subramaniam D, Thombre R, Dhar A, Anant S (2014) DNA Methyltransferases: a novel target for prevention and therapy. Front Oncol 4 10.3389/fonc.2014.00080 PMC4013461 24822169

[CR149] Subramanian I, Verma S, Kumar S, Jere A, Anamika K (2020). Multi-omics data integration, interpretation, and its application. Bioinform Biol Insights.

[CR150] Sung H, Ferlay J, Siegel RL, Laversanne M, Soerjomataram I, Jemal A (2021). Global Cancer Statistics 2020: GLOBOCAN estimates of incidence and mortality worldwide for 36 cancers in 185 countries. CA A Cancer J Clin.

[CR151] Suter RK, Rodriguez-Blanco J, Ayad NG (2020). Epigenetic pathways and plasticity in brain tumors. Neurobiol Dis.

[CR152] Swan AL, Stekel DJ, Hodgman C, Allaway D, Alqahtani MH, Mobasheri A (2015). A machine learning heuristic to identify biologically relevant and minimal biomarker panels from omics data. BMC Genomics.

[CR153] Tan WCC, Nerurkar SN, Cai HY, Ng HHM, Wu D, Wee YTF (2020). Overview of multiplex immunohistochemistry/immunofluorescence techniques in the era of cancer immunotherapy. Cancer Commun.

[CR154] Tanioka T, Hattori A, Masuda S, Nomura Y, Nakayama H, Mizutani S (2003). Human leukocyte-derived arginine aminopeptidase. J Biol Chem.

[CR155] The Cancer Genome Atlas Research Network (2014). Comprehensive molecular characterization of gastric adenocarcinoma. Nature.

[CR156] Theofilopoulos AN, Kono DH, Baccala R (2017). The multiple pathways to autoimmunity. Nat Immunol.

[CR157] Thorsson V, Gibbs DL, Brown SD, Wolf D, Bortone DS, Ou Yang T-H (2018). The immune landscape of cancer. Immunity.

[CR158] Tirapu I, Huarte E, Guiducci C, Arina A, Zaratiegui M, Murillo O (2006). Low surface expression of B7-1 (CD80) is an immunoescape mechanism of colon carcinoma. Cancer Res.

[CR159] Toki T, Itoh J, Kitazawa J, Arai K, Hatakeyama K, Akasaka J (1997). Human small Maf proteins form heterodimers with CNC family transcription factors and recognize the NF-E2 motif. Oncogene.

[CR177] van Elsas MJ, van Hall T, van der Burg SH (2020) Future challenges in cancer resistance to immunotherapy. Cancers 12:935 10.3390/cancers12040935 PMC7226490 32290124

[CR160] Villanueva L, Álvarez-Errico D, Esteller M (2020). The contribution of epigenetics to cancer immunotherapy. Trends Immunol.

[CR162] Waldman AD, Fritz JM, Lenardo MJ (2020). A guide to cancer immunotherapy: from T cell basic science to clinical practice. Nat Rev Immunol.

[CR163] Wang X, Li J, Dong K, Lin F, Long M, Ouyang Y (2015). Tumor suppressor miR-34a targets PD-L1 and functions as a potential immunotherapeutic target in acute myeloid leukemia. Cell Signal.

[CR164] Wang Y, Wang L (2017). miR-34a attenuates glioma cells progression and chemoresistance via targeting PD-L1. Biotechnol Lett.

[CR165] Wienand K, Chapuy B, Stewart C, Dunford AJ, Wu D, Kim J (2019). Genomic analyses of flow-sorted Hodgkin Reed-Sternberg cells reveal complementary mechanisms of immune evasion. Blood Adv.

[CR166] Woods DM, Sodré AL, Villagra A, Sarnaik A, Sotomayor EM, Weber J (2015). HDAC Inhibition Upregulates PD-1 Ligands in Melanoma and Augments Immunotherapy with PD-1 Blockade. Cancer Immunol Res.

[CR167] Yokosuka T, Saito T (2010) The immunological synapse, TCR microclusters, and T cell activation. In: Saito T, Batista FD (eds) Immunological synapse, current topics in microbiology and immunology, vol 340. Springer Berlin Heidelberg, Berlin, Heidelberg, p 81–107 10.1007/978-3-642-03858-7_5 19960310

[CR168] Zerdes I, Matikas A, Bergh J, Rassidakis GZ, Foukakis T (2018). Genetic, transcriptional and post-translational regulation of the programmed death protein ligand 1 in cancer: biology and clinical correlations. Oncogene.

[CR169] Zhang H, Dai Z, Wu W, Wang Z, Zhang N, Zhang L (2021). Regulatory mechanisms of immune checkpoints PD-L1 and CTLA-4 in cancer. J Exp Clin Cancer Res.

[CR170] Zhang W, Kong X, Ai B, Wang Z, Wang X, Wang N (2021). Research progresses in immunological checkpoint inhibitors for breast cancer immunotherapy. Front Oncol.

[CR171] Zhang Y, Ling C (2018). A strategy to apply machine learning to small datasets in materials science. npj Comput Mater.

[CR172] Zhang J, Yang C, Wu C, Cui W, Wang L (2020). DNA methyltransferases in cancer: biology, paradox, aberrations, and targeted therapy. Cancers.

[CR173] Zhang Y, Zhang Z (2020). The history and advances in cancer immunotherapy: understanding the characteristics of tumor-infiltrating immune cells and their therapeutic implications. Cell Mol Immunol.

[CR174] Zhao X, Ji Z, Xie Y, Liu G, Li H (2017). MicroRNA-154 as a prognostic factor in bladder cancer inhibits cellular malignancy by targeting RSF1 and RUNX2. Oncol Rep.

[CR175] Zingg D, Arenas-Ramirez N, Sahin D, Rosalia RA, Antunes AT, Haeusel J (2017). The Histone methyltransferase Ezh2 controls mechanisms of adaptive resistance to tumor immunotherapy. Cell Rep.

[CR176] Zurawek M, Dzikiewicz-Krawczyk A, Izykowska K, Ziolkowska-Suchanek I, Skowronska B, Czainska M (2018). miR-487a-3p upregulated in type 1 diabetes targets CTLA4 and FOXO3. Diabetes Res Clin Pract.

